# Acceptability and Feasibility of a 13-Week Pilot Randomised Controlled Trial Testing the Effects of Incremental Doses of Beetroot Juice in Overweight and Obese Older Adults

**DOI:** 10.3390/nu13030769

**Published:** 2021-02-26

**Authors:** Abrar M. Babateen, Oliver M. Shannon, Gerard M. O’Brien, Edward Okello, Anmar A. Khan, Sofia Rubele, Emma Wightman, Ellen Smith, Nicholas McMahon, Dilara Olgacer, Christina Koehl, William Fostier, Inês Mendes, David Kennedy, John C. Mathers, Mario Siervo

**Affiliations:** 1 Human Nutrition Research Centre, Population Health Sciences Institute, Newcastle University, Newcastle Upon Tyne NE2 4HH, UK; A.m.o.babateen2@ncl.ac.uk (A.M.B.); oliver.shannon@ncl.ac.uk (O.M.S.); edward.okello@newcastle.ac.uk (E.O.); olgacerdilara@gmail.com (D.O.); chrissi.koechl18@gmail.com (C.K.); W.fostier@ncl.ac.uk (W.F.); john.mathers@ncl.ac.uk (J.C.M.); 2 Faculty of Applied Medical Sciences, Clinical Nutrition Department, Umm Al-Qura University, Makkah 21421, Saudi Arabia; aaakhan@uqu.edu.sa; 3 Translational and Clinical Research Institute, Newcastle University, Newcastle Upon Tyne NE1 7RU, UK; 4 Department of Geriatrics, St Bortolo Hospital, Vicenza, 37030 Verona, Italy; sofiarubele89@hotmail.it; 5 Brain Performance and Nutrition Research Centre, Northumbria University, Newcastle Upon Tyne NE1 8ST, UK; emma.l.wightman@northumbria.ac.uk (E.W.); ellen.smith@northumbria.ac.uk (E.S.); david.kennedy@northumbria.ac.uk (D.K.); 6 Nutrition Trials at Northumbria (NUTRAN), Northumbria University, Newcastle Upon Tyne NE1 8ST, UK; 7 School of Human Movement and Nutrition Sciences, University of Queensland, St. Lucia, QLD 4067, Australia; n.mcmahon2@uq.edu.au; 8 Endocrinology and Nutrition Department, Divino Espirito Santo Hospital, D. Manuel I Avenue, 9500-370 Azores, Portugal; ines_c_mendes@hotmail.com; 9 School of Life Sciences, Queen’s Medical Centre, The University of Nottingham Medical School, Nottingham NG7 2UH, UK

**Keywords:** dietary nitrate, older adults, overweight/obese

## Abstract

Nitrate-rich food can increase nitric oxide production and improve vascular and brain functions. This study examines the feasibility of a randomised controlled trial (RCT) testing the effects of prolonged consumption of different doses of dietary nitrate (NO_3_^−^) in the form of beetroot juice (BJ) in overweight and obese older participants. A single-blind, four-arm parallel pilot RCT was conducted in 62 overweight and obese (30.4 ± 4 kg/m^2^) older participants (mean ± standard deviation (SD), 66 ± 4 years). Participants were randomized to: (1) high-NO_3_^−^ (HN: 2 × 70 mL BJ/day) (2) medium-NO_3_^−^ (MN: 70 mL BJ/day), (3) low-NO_3_^−^ (LN: 70 mL BJ on alternate days) or (4) Placebo (PL: 70 mL of NO_3_^−^-depleted BJ on alternate days), for 13 weeks. Compliance was checked by a daily log of consumed BJ, NO_3_^−^ intake, and by measuring NO_3_^−^ and NO_2_^−^ concentrations in plasma, saliva, and urine samples. Fifty participants completed the study. Self-reported compliance to the interventions was >90%. There were significant positive linear relationships between NO_3_^−^ dose and the increase in plasma and urinary NO_3_^−^ concentration (R^2^ = 0.71, *p* < 0.001 and R^2^ = 0.46 *p* < 0.001, respectively), but relationships between NO_3_^−^ dose and changes in salivary NO_3_^−^ and NO_2_^−^ were non-linear (R^2^ = 0.35, *p* = 0.002 and R^2^ = 0.23, *p* = 0.007, respectively). The results confirm the feasibility of prolonged BJ supplementation in older overweight and obese adults.

## 1. Introduction

Increased life expectancy is associated with a concomitant rise in the occurrence of age-related medical conditions, including cardiovascular, metabolic and neurological diseases [1]. In the UK, the number of individuals aged 60 years and over is expected to increase by 8.6 million over the next five decades [2]. Therefore, it is of paramount importance to promote and protect the quality of life of older people through developing primary preventive interventions. Since diet has a major influence on ageing and on the risk of age-related diseases [3], the identification of nutritional and lifestyle factors associated with healthy ageing is a priority. However, conducting nutritional intervention studies in older populations is often challenging.

Recruitment of older participants in clinical research studies can be demanding [4]. Older participants are more likely to be excluded from studies due to pre-existing medical conditions, such as limited mobility, polypharmacy or the presence of chronic diseases [5]. Hence, recruitment may become a more difficult and time-consuming process despite the fact that older participants are often more willing to participate in clinical trials compared with younger participants [6]. In addition, given the medical complexities associated with ageing, attrition rates from studies may be increased, which is another concern from the researcher’s perspective [7]. Compliance with dietary interventions or study protocols may be difficult for older people. The potential for adverse events could also affect compliance as well as difficulties around high frequency of intake, rigid conditions for ingestion times, and onerous preparation of meals in study protocols [8]. Although some strategies to address reasons for non-participation in clinical research and to improve compliance with study protocols have been proposed [7,9,10], there is considerable need for improved design and performance of intervention studies in older people. This gap can be addressed through sharing of experiences, challenges and successes in recruiting and retaining older adults in nutritional intervention studies and related clinical research.

This pilot randomised controlled trial (RCT) was designed to determine the feasibility and acceptability of the protocol for a 13-week intervention study in which overweight and obese older participants were asked to consume different doses of NO_3_^−^ rich beetroot juice (BJ). Ageing and obesity are associated with reduced NO availability due to several factors including increased oxidative stress, decreased activity of endothelial NO synthase and also increased production of endogenous NO inhibitors such as asymmetric dimethyl arginine (ADMA) [11]. Thus, supplemental NO_3_^−^ may be more effective in older, obese individuals than in healthy younger people. Whilst a number of studies have investigated the effects of BJ in older adults [12,13,14], these studies have been characterised by short duration which may pose fewer issues with compliance than longer-term studies. To our knowledge, the present 13-week study is one of the longest RCTs that has tested the effect of different BJ doses in older overweight and obese participants. 

## 2. Materials and Methods

### 2.1. Ethical Approval

The study was approved by the Faculty of Medical Sciences, Newcastle University (1503/4477/2018). The intervention study was registered with the ISRCTN registry (International Standard Randomised Controlled Trial Number) (ISRCTN14746723).

### 2.2. Participants and Study Design

This was a randomised, single-blind, placebo-controlled, four-arm parallel feasibility trial. All recruited participants were older (60 to 75 years), overweight or obese (body mass index (BMI)) range: 25–40 kg/m^2^) adults. Participants were recruited from members of staff or the general public who responded to our newspaper or social media advertisements, flyers or emails between July 2018 and April 2019. Eligible participants were screened to confirm their eligibility and written informed consent was obtained subsequently from them before entering the study. Participants were randomised to one of four treatments that were administered for 13 weeks. The treatments were: (1) High Nitrate (HN); two 70 mL shots of concentrated BJ per day (~400 mg of NO_3_^−^ per shot), one in the morning (~8:00am) and one in the evening (~9:00pm). (2) Medium Nitrate (MN), one shot of concentrated BJ in the evening (~9:00pm). (3) Low Nitrate (LN), one shot of concentrated BJ every other evening (~9:00pm). (4) Placebo (PL), one shot of NO_3_^−^ depleted BJ (~0.001 mg of NO_3_^−^) every other evening (~9:00pm). The randomization pattern was generated using the RAND function in Excel (Excel Microsoft software, Version 16.46, Microsoft corp., Redmond, WA, USA). Further details on participants recruitment, selection, and screening have been described elsewhere [15].

### 2.3. Primary and Secondary Outcome Measures

The primary outcomes of the trial were measures of feasibility, acceptability, and compliance with the interventions and with the study protocol. Secondary outcomes included testing the effects of the different doses of dietary NO_3_^−^ on measures of cognitive, vascular and pulmonary functions and on cerebral blood flow.

### 2.4. Data Collection Procedures

All data were collected over one year between July 2018 and July 2019. Baseline measurements were taken on two separate consecutive days, as some measurements were conducted in two different locations (Newcastle University and Northumbria University). Participants were asked to follow a 24-h run-in diet to standardise their NO_3_^−^ intake which included avoidance of green leafy vegetables and avoiding alcohol and caffeine consumption for 24 h before both baseline visits. On the first day, participants arrived at the research facility in the morning (between 09:30–10:00) in a fasted condition (~12 h after the previous meal). Body composition, waist circumference and biological samples including blood, saliva and urine samples, as well as salivary strips, were collected. In addition, measurements of cognitive, vascular and pulmonary functions were performed. The measurement protocols have been described [15] and the results of the secondary outcome measures will be published in due course.

Participants returned the following day for the second baseline visit at Northumbria University between 15:00 and 16:00 to perform the cerebral blood flow measurement as described elsewhere [15]. Biological samples including salivary NO_2_^−^ strips, saliva and urine samples were collected in the morning at home after an overnight fast (~12 h).

The end study visits were performed after 13 weeks following the same order as for the baseline measurements. These two visits were scheduled to occur ~12 h after the consumption of the last BJ dose for HN and MN groups, and ~36 h after the consumption of the last BJ dose for the LN and PL groups.

Participants were asked to complete the short-version of the international physical activity questionnaire (IPAQ) [16] at home to estimate their physical activity over the week prior to the first baseline, the interim (6 weeks) and the end of the study (13 weeks). To assess dietary NO_3_^−^ intake (excluding the intervention supplement) during the study, participants were asked to record their food intake every two weeks using an online 24-h dietary recalls (Intake24; six dietary records were collected in total). Participants were instructed not to change their dietary habits or physical activity patterns and to avoid using mouthwash during the study as the latter has been shown to destroy oral bacteria involved in NO_3_^−^ reduction in the body [17]. All participants were informed that reasonable travel expenses would be covered, and each participant received a £60 voucher at the end of the study.

### 2.5. Compliance

#### 2.5.1. Compliance with the Dietary Intervention

**Figure 1:**
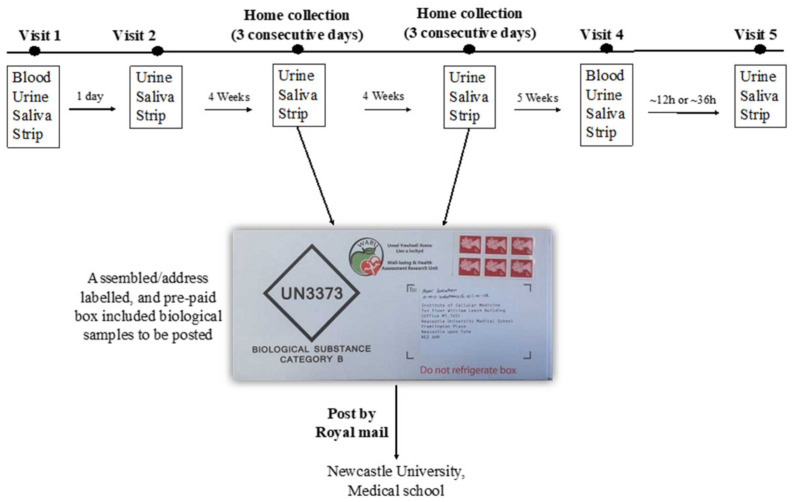
Summary of the collection of biological samples during the study. Urine collection kit used is (Biological substance Category B, Shuttlepac, UK).

The assessment of participant compliance with the intervention has been described in detail elsewhere [15]. Briefly, compliance was checked primarily by completing a daily log to record the time of BJ consumption, and also to report any BJ bottles that were not consumed. Compliance with the intervention was also assessed objectively by measuring NO_3_^−^ and NO_2_^−^ concentrations in plasma, saliva and urine samples and using salivary strips. The protocol for the collection of the biological samples is described in Figure 1.

Blood samples were collected in the research centre at baseline and after 13 weeks (visit 1 and visit 4). Urine, saliva and salivary NO_2_^−^ strips were collected at baseline, at 4 weeks (for 3 consecutive days), 8 weeks (for 3 consecutive days), and 13 weeks.

#### 2.5.2. Compliance with the Study Protocol

Participants were requested to collect biological samples (urine and saliva), and salivary NO_2_^−^ strips at home and to post them to the research centre using the national mail delivery service. In addition, participants were asked to report their dietary intake using Intake24 every 2 weeks.

### 2.6. Recording Food Intake Using Intake24

Intake24 is a user-friendly, interactive web-based tool based on 24-h recall for the assessment of dietary intake [18], that was used to assess NO_3_^−^ intake every two weeks during the study (6 entries in total for each participant). In this manuscript, the number of 24-h records completed at each time point is reported.

### 2.7. Collection and Analysis of Biological Samples

Samples collection procedures have been described in detail elsewhere [15]. Briefly, blood samples were collected after an overnight fast (~12 h) at baseline and 13 weeks. The samples were processed within 10 min of collection to minimize NO_2_^−^ degradation. Salivary NO_2_^−^ strips, urine and saliva samples were collected at baseline (both visits) and then again at 4 weeks (3 consecutive days), 8 weeks (3 consecutive days), and 13 weeks of intervention (both visits) (see Figure 1). Blood and salivary samples were used to measure NO_3_^−^ and NO_2_^−^ concentrations, while urine samples were used to measure NO_3_^−^ concentrations. Salivary strips (Berkeley Test strips, Berkeley life, Chicago, IL, USA) were used to estimate salivary NO_2_^−^ concentration and also to evaluate their utility as a measure of longer-term compliance to NO_3_^−^ interventions.

Sample analysis was conducted using ozone-based chemiluminescence (Sievers NOA 280i, Analytix Ltd., Durham, UK), as previously described [19]. Salivary NO_2_^−^ strips analysis was conducted as described [15]. A brief description of both methods is reported in the online Supplementary Materials.

### 2.8. Acceptability with Intervention and Study Protocol

At the end of the study, participants completed a 25-item questionnaire to obtain feedback on the intervention and study protocol. The questionnaire took 10–15 min to complete. Questions were designed to obtain information on the following specific topics: (1) reasons for joining the study and expectation from the study, (2) the duration of the study, (3) nutritional supplementation and (4) measurement protocols. The questionnaire included a range of closed and open questions. Based on the participants’ responses to the open questions, the answers were categorised into specific topics and the frequency of each response topic was calculated (See Supplementary Materials).

### 2.9. Sample Size Calculation

This was a pilot study designed to assess the feasibility and acceptability of the proposed intervention. A sample size of 15 per intervention group was based on (i) the predicted effect size of the intervention on cognitive changes (Trail making test-B) [20] and (ii) the guidelines indicated by Whitehead et al., (2016) [21], who provided guidance on sample size calculation for pilot studies with the aim of maximising use of resources and avoiding type II errors. Specifically, a sample size of 15 individuals per group would provide a 90% power to detect a medium effect size between 0.3 and 0.7.

### 2.10. Data Presentation and Statistical Analysis

Continuous data are summarised as mean ± standard deviation (SD) and categorical data are presented as percentage (%). Normality distribution of data was checked by visual inspections of histograms and by Shapiro-Wilks test. One-way analysis of variances (ANOVA) was used to compare baseline characteristics between intervention groups. Categorical variables were analysed by Chi-square test.

Feasibility and compliance: Feasibility of the intervention was evaluated by collating quantitative information on eligibility, recruitment and retention of participants in the trial. Retention was estimated by calculating the proportion of enrolled participants who dropped out and were lost to follow up. Intervention compliance rate was estimated as the proportion of BJ shots consumed relative to the total dispensed shots i.e., (number of BJ bottles consumed/total number of BJ required to be consumed during the 13 weeks) X 100. Participants were considered compliant if they reported consumption of 80% or more of their BJ during the 13 weeks of the study. Compliance with the intervention was also assessed by measuring changes in biomarkers of dietary NO_3_^−^ intake including plasma, urinary and salivary concentrations of NO_3_^−^ and NO_2_^−^. Compliance with other aspects of the study protocol including home collection of biological samples and posting the samples to the laboratory, and recording dietary intakes using Inake24 was evaluated.

Biomarkers analysis: Summary data are presented as mean ± standard error of the mean (SEM) in figures, and 95% confidence intervals (CI) in the text. Only data from participants who completed the study were included in the analysis. A paired *t*-test was used to assess the between-day repeatability of biomarkers for the two visits conducted at baseline and also at the end of the study. To evaluate changes in biomarker concentrations post-supplementation, the mean value for both testing days (baseline and end of the study) was used in the analyses. Changes over time were analysed using two-factor repeated-measure ANOVA with time as a within-subjects factor and intervention as a between-subjects factor. Models were checked for sphericity assumptions and multivariate models were applied if assumptions were violated. If the model was significant, Dunnett’s test was used to compare the effects of different doses of NO_3_^−^. In addition, the change from baseline was calculated for each individual and then treatments were compared using one-way ANOVA. One-way ANOVA was also used to determine whether delays in the delivery of the samples collected at home affected salivary and urinary NO_3_^−^ and NO_3_^−^ concentrations (all interventions were combined together in this analysis). To investigate dose-response relationships between supplemental doses of NO_3_^−^ and concentrations of NO_3_^−^ and NO_2_^−^ in biofluids, polynomial regression analysis was used. Associations between salivary NO_2_^−^ concentrations measured by chemiluminescence and the values obtained from salivary strips were investigated using Pearson’s correlation analysis. Statistical significance was set at *p* < 0.05. All statistical analyses were completed using (IBM SPSS, version 23, New York, NY, USA).

## 3. Results

### 3.1. Baseline Characteristics

The baseline characteristics (summarised in Table 1) show that the participants in each intervention group were well-matched for anthropometric variables, age and blood pressure. The age of participants ranged from 60 to 73 years (mean ± SD, 66 ± 4) and 62% were men (*n* = 38). Fifty-six percent of participants were overweight and the remaining 44% were obese. BMI ranged from 25 to 39 kg/m^2^ (30.4 ± 4 kg/m^2^). Systolic blood pressure ranged from 110 to 167 mmHg (135 ± 15 mmHg) and diastolic blood pressure from 60 to 100 mmHg (77 ± 10 mmHg). Six participants were hypertensive and on antihypertensive medication and 27 participants were on other medications as reported in Table 1.

### 3.2. Recruitment and Retention

**Figure 2:**
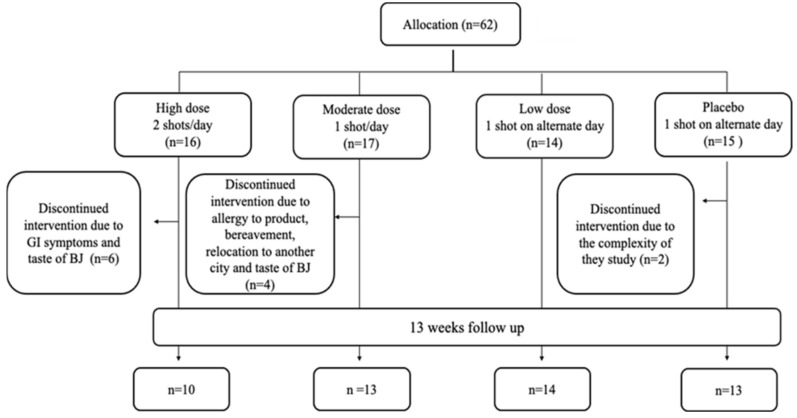
Flowchart of participants recruited into the feasibility study.

Sixty-two participants were included in the study and randomized to one of the four intervention groups as follows: 16 participants were allocated to Group 1 (2 shots of BJ/day, every morning and evening), 17 participants to Group 2 (1 shots of BJ/day, every evening), 14 participants to Group 3 (1 shot of BJ every other evening) and 15 participants were allocated to Group 4 (Placebo, 1 shot of BJ depleted NO_3_^−^ every other evening) (please see [15] for further details). The overall attrition rate for the study was 19% with 12 participants dropping from the study. The highest attrition rate was found to be in the HN group with 38% of participants who enrolled to this group, followed by the MN and PL groups with 24% and 20%, respectively. No participants dropped out from LN group. Fifty participants completed the study. Figure 2 shows the flowchart of participants recruited into the feasibility study. Reasons for withdrawal from the study are summarised in Table 2.

### 3.3. Compliance

#### 3.3.1. Intervention Intake

The daily log sheets used to record BJ consumption indicated excellent compliance with the intervention for the large majority of participants. Across all intervention groups, the overall compliance was more than 90%. The compliance rate was similar between groups (*p* = 0.52). The compliance with intervention for each individual participant is summarised in Figure S1.

#### 3.3.2. Sample Collection at Home and Delivery by Post to the Research Centre

All participants who completed the study (*n* = 50) adhered to the protocol for home collection of the biological samples during the study. Participants did not report any difficulties with posting the samples using the pre-paid delivery boxes. We expected to receive the samples within 2 days from posting and we asked participants specifically to avoid posting the samples during weekends or on public holidays. However, we experienced delays in receiving some of the samples. In total, 16 samples were delivered at least 3 days after being posted. The number of days between posting and receipt of the samples at the research centre are detailed in Figure S2. During the first month of the study, 22 boxes were received the next day after posting, 8 were received after 2 days, and 8 boxes were delivered at least 3 days after posting. During the second month of the study, 12 boxes were received the next day after posting, 13 boxes were received after 2 days, and 8 boxes were received at least 3 days after posting. Some samples were also collected from the participant’s home (*n* = 5), and in some cases participants took the samples directly to the research centre (*n* = 28).

#### 3.3.3. Recording Dietary Intake Using Intake24

Thirty-eight (76%) participants completed all 6 dietary intake records. Of those, 3 participants reported their intakes on paper due to difficulty with accessing the software, and dietary data were then uploaded to Intake24 by a member of the research team. Twelve (24%) participants had incomplete dietary intake records due to non-adherence. Among that group, 9 participants had less than three dietary records. This finding is summarised in Figure S3.

### 3.4. Biomarkers of Nitrate Intake

#### 3.4.1. Plasma Nitrate and Nitrite Concentrations

**Figure 3:**
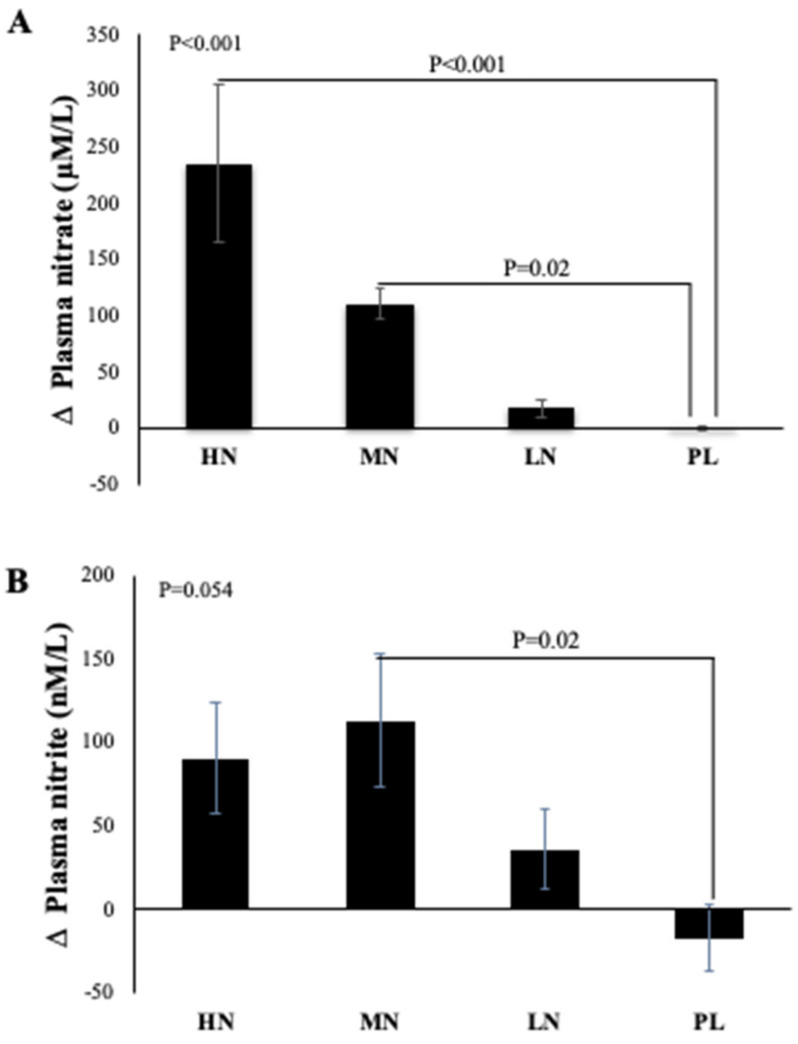
Mean changes in plasma NO_3_^−^ (**A**) and NO_2_^−^ (**B**) concentrations of incremental doses of dietary NO_3_^−^ in form of BJ in older overweight and obese adults. HN (High NO_3_^−^; two 70 mL shots of BJ/day, morning and evening), MN (Medium NO_3_^−^; 70 mL of BJ/day), LN (Low NO_3_^−^; 70 mL of BJ every alternate days) and PL (placebo; 70 mL of NO_3_^−^ depleted BJ). Each shot of BJ contains 400 mg of NO_3_^−^. The change at 13-weeks from baseline data (**A**,**B**) were analysed with one-way ANOVA. Data are expressed as mean ± standard error of the mean (SEM) (*n* = 49).

Due to difficulty in collecting samples from one participant, data from 49 participants were included in the analysis. One-way ANOVA reveals that there was a statistically significant difference between intervention groups in the change from baseline in plasma NO_3_^−^ concentration at 13-weeks (*p* < 0.001). Compared with PL, plasma NO_3_^−^ concentrations increased significantly after HN (Δ 236 µM/L, 95% CI: 126, 346 µM/L, *p* < 0.001) and MN (Δ 111 µM/L, 95% CI: 10, 212 µM/L, *p* = 0.02) doses. No significant differences were found between the LN and PL doses (Δ 18 µM/L, 95% CI: -82, 119 µM/L, *p* = 0.94) (Figure 3A). However, evidence for a change from baseline in plasma NO_2_^−^ concentration was less convincing (*p* = 0.054). Relative to PL, plasma NO_2_^−^ was significantly increased only after the MN dose (Δ 130 µM/L, 95% CI: 12, 248µM/L, *p* = 0.02). There were no significant differences in the change from baseline for plasma NO_2_^−^ for the HN (*p* = 0.12) or LN (*p* = 0.56) doses compared with PL (Figure 3B).

**Figure 4:**
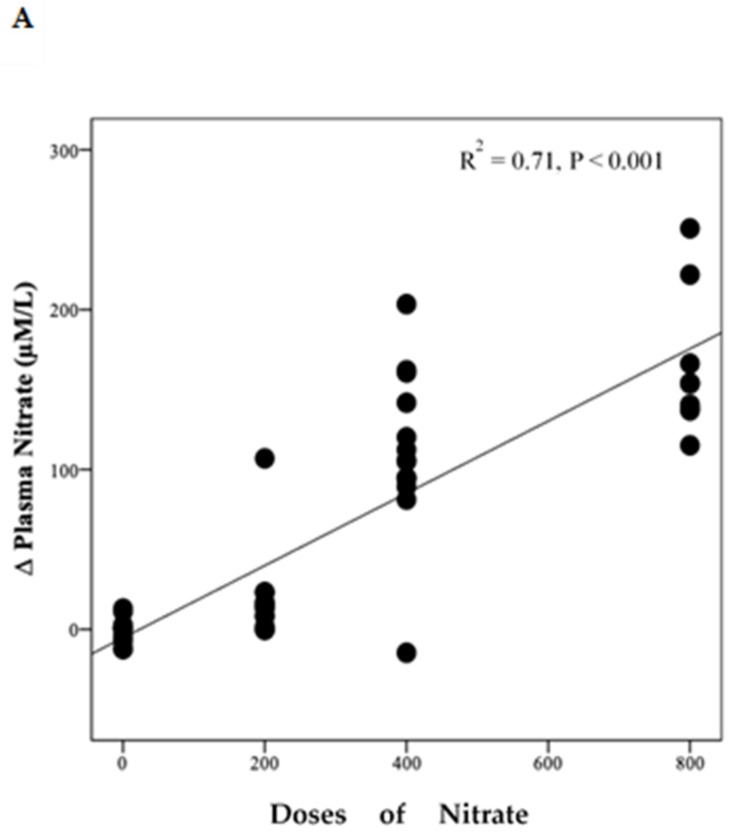

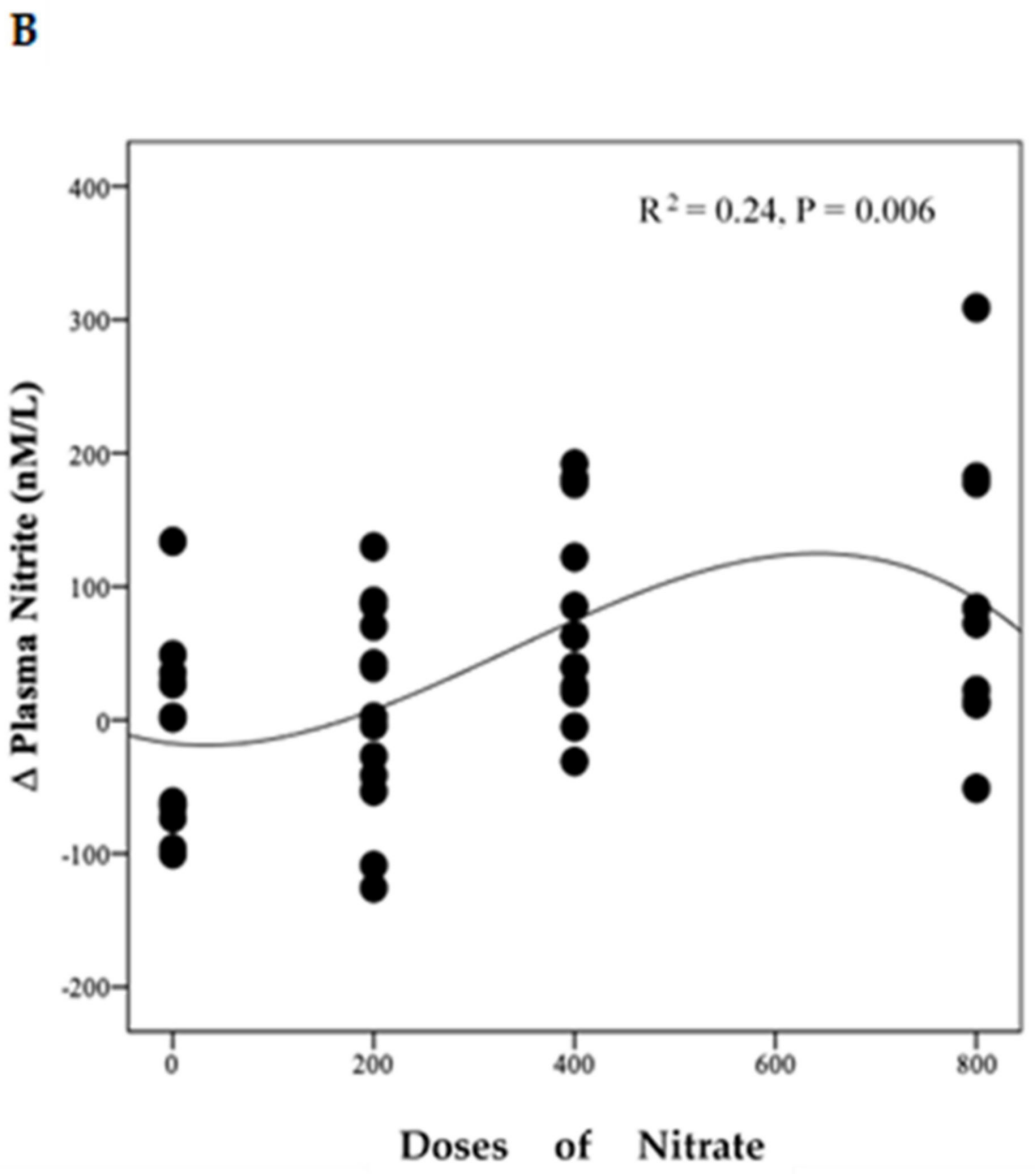
Relationship between changes in plasma NO_3_^−^, NO_2_^−^ and doses of NO_3_^−^. This figure shows the relationship between changes in plasma NO_3_^−^ (**A**) and NO_2_^−^ (**B**) and doses of NO_3_. Regression lines are fitted to each distribution, (**A**) is linear and (**B**) is cubic, (*n* = 49).

Polynomial regression analysis revealed a significant positive linear relationship between NO_3_^−^ dose and the change in plasma NO_3_^−^ concentration (R^2^ = 0.71, *p* < 0.001) (Figure 4A). However, there was a non-linear relationship (cubic model) between NO_3_^−^ doses and the change from baseline in plasma NO_2_^−^ concentration (R^2^ = 0.24, *p* = 0.006) (Figure 4B).

#### 3.4.2. Salivary Nitrate and Nitrite Concentrations

**Figure 5:**
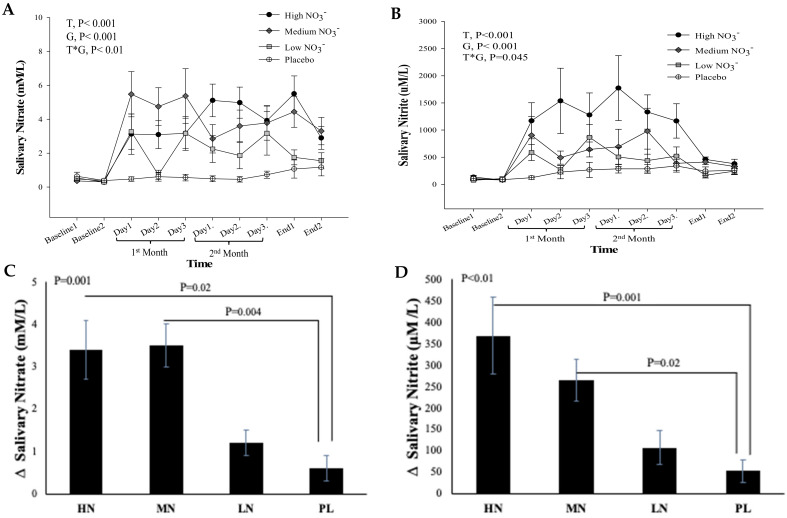
Salivary NO_3_^−^ and NO_2_^−^ concentrations in older overweight and obese adults. (**A**,**B**) Mean salivary NO_3_^−^ and NO_2_^−^ concentrations measured at baseline, 4 weeks, 8 weeks, and after 13-weeks for each of the intervention groups, respectively. (**C**,**D**) The mean change in salivary NO_3_^−^ and NO_2_^−^ concentrations between baseline and week 13 for each if the intervention groups, respectively. HN (High NO_3_^−^; two 70 mL shots of BJ/day, morning and evening), MN (Medium NO_3_^−^; 70 mL of BJ/day), LN (Low NO_3_^−^; 70 mL of BJ every alternate days) and PL (placebo; 70 mL of NO_3_^−^ depleted BJ). Each shot of BJ contains 400 mg of NO_3_^−^ (**A**,**B**) Data were analysed using a 2-factor repeated measures ANOVA (time*group of intervention). (**C**,**D**) These changes of from baseline were analysed with one-way ANOVA. Data are expressed as mean ± SEM, (*n* = 50).

There was no significant difference between and within the intervention groups in salivary NO_3_^−^ and NO_2_^−^ concentrations measured at the two baseline visits (*p* = 0.29, *p* = 0.6, respectively), confirming that participants complied with the instruction to maintain a low NO_3_^−^ diet before both baseline visits (Figure 5A,B). For salivary NO_3_^−^, repeated measures analysis showed a significant effect of time (*p* < 0.001), intervention (*p* < 0.001), and a significant time*intervention interaction (*p* = 0.01) (Figure 5A). There was no significant difference in salivary NO_3_^−^ concentrations between the two end-of-study measurements within the intervention groups (*p* > 0.05). One-way ANOVA revealed a statistically significant difference between intervention groups in the change from baseline in salivary NO_3_^−^ at 13 weeks (*p* < 0.001). Compared with PL, salivary NO_3_^−^ concentration at 13 weeks significantly increased after HN and MN (Δ 2.7 µM/L, 95% confidence interval (CI): 0.4, 4.9, *p* = 0.02), (Δ 2.9 µM/L, 95% CI: 0.8, 5.0, *p* = 0.004), respectively, but there was no significant difference between LN dose and PL (*p* = 0.30) (Figure 5C). 

For salivary NO_2_^−^, a significant effect of time (*p* < 0.001) and intervention (*p* < 0.001), and a significant interaction term (*p* = 0.045) was revealed by repeated measures analysis. Similar to salivary NO_3_^−^, no significant difference was detected between the two end of study measurements within the intervention groups (*p* > 0.05) (Figure 5B). There was a statistically significant difference between intervention groups in the change from baseline in salivary NO_2_^−^ at 13 weeks (*p* = 0.001). Salivary NO_2_^−^ concentration at 13 weeks increased significantly after HN and MN (Δ 319 µM/L, 95% CI: 119, 520 µM/L, *p* = 0.001), (Δ 217 µM/L, 95% CI: 34, 401 µM/L, *p* = 0.02), respectively, compared with PL (Figure 5D). No significant difference was observed between LN dose and PL (*p* = 0.43).

**Figure 6:**
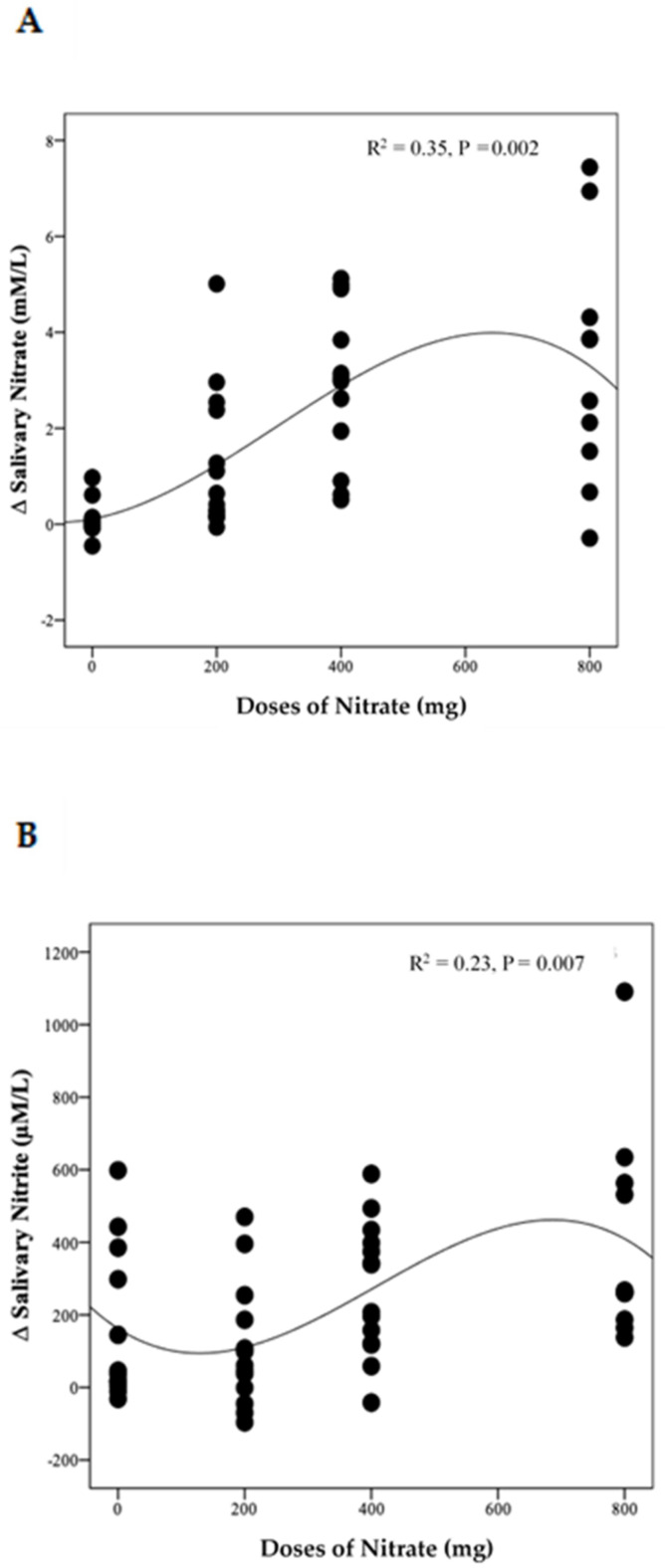
The relationship between the changes in salivary NO_3_^−^ (**A**) and NO_2_^−^ (**B**) and daily doses of NO_3_^−^ delivered during the intervention. A cubic model was the best fit for each distribution (*n* = 50).

The distinct pattern of changes in concentrations of NO_3_^−^ and NO_2_^−^ for the LN group becomes even clearer when data from week 4 and week 8 are combined (Figure S4A,B). This showed a significant difference between time points for both NO_3_^−^ and NO_2_^−^ (*p* = 0.003 and 0.002, respectively). Compared with baseline, salivary NO_3_^−^ concentrations increased significantly on the 1st and 3rd day of collection (based on combined data obtained at week 4 and week 8) (Δ 2.3 mM/L, 95% CI: 0.5, 4.2 mM/L, *p* = 0.009) and (Δ 2.8 mM/L, 95% CI: 0.9, 4.6 mM/L, *p* = 0.002), respectively. Similarly, salivary NO_2_^−^ concentrations increased significantly on the 1st and 3rd days ((Δ 454.8 µM/L, 95% CI: 65.2, 844.5 µM/L, *p* = 0.02) and (Δ 587.7 µM/L, 95% CI: 198.0, 977.4 µM/L, *p* = 0.001), respectively. No significant difference was found between data collected on the 2nd day, at baseline and end of the study for both salivary NO_3_^−^ and NO_2_^−^ concentrations (*p* > 0.05). There was a significant non-linear relationship between NO_3_^−^ doses and changes in salivary NO_3_^−^ (R^2^ = 0.35, *p* = 0.002) and NO_2_^−^ (R^2^ = 0.23, *p* = 0.007) concentrations (Figure 6A,B)

#### 3.4.3. Urinary Nitrate Concentration

**Figure 7:**
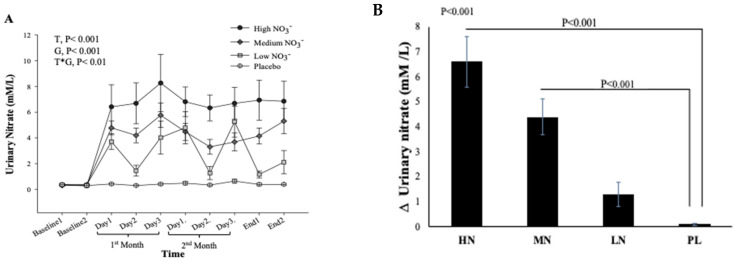
Urinary NO_3_^−^ concentrations in older overweight and obese adults. (**A**) Mean urinary NO_3_^−^ concentrations measured at baseline, 4 weeks, 8 weeks, and after 13-weeks for each of the intervention groups. (**B**) The Mean of the change in urinary NO_3_^−^ concentrations between baseline and week 13 for each of the intervention groups. HN (High NO_3_^−^; two 70 mL shots of BJ/day, morning and evening), MN (Medium NO_3_^−^; 70 mL of BJ/day), LN (Low NO_3_^−^; 70 mL of BJ every alternate days) and PL (placebo; 70 mL of NO_3_^−^ depleted BJ). Each shot of BJ contains 400 mg of NO_3_^−^. (**A**) Data were analysed using a 2-factor repeated measures ANOVA (time*group of intervention). (**B**) These changes from baseline were analysed with one-way ANOVA. Data are expressed as mean ± SEM, (*n* = 50).

**Figure 8:**
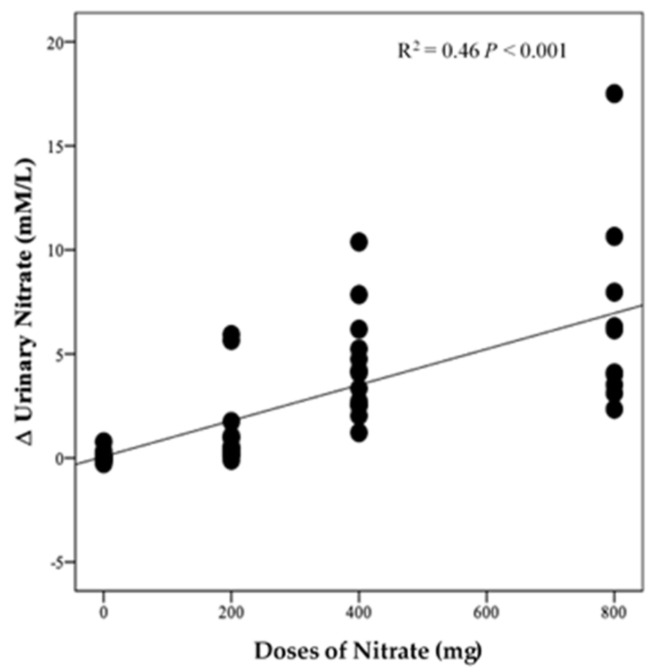
Linear relationship between the changes in urinary NO_3_^−^ and doses of NO_3_^−^ (*n* = 50).

There was no significant difference in urinary NO_3_^−^ concentrations at baseline between intervention groups (*p* = 0.35, Figure 7A). However, there was a significant effect of time (*p* < 0.001), intervention (*p* < 0.001), and time*intervention interaction for urinary NO_3_^−^ (*p* < 0.001) and one-way ANOVA revealed a statistically significant difference between intervention groups in the change from baseline in urinary NO_3_^−^ at 13 weeks (*p* < 0.001). Compared with PL, urinary NO_3_^−^ concentration was significantly greater after HN (Δ 6.5 µM/L, 95% CI: 3.8, 9.2, *p* < 0.001) and MN (Δ 4.3 µM/L, 95% CI: 1.8, 6.8, *p* < 0.001) (Figure 7B). Figure 7A shows that urinary NO_3_^−^ concentrations were high and stable over time with HN and MN doses whereas urinary NO_3_^−^ concentrations were relatively unchanged in the PL group. The pattern of change in urinary NO_3_^−^ concentration for the LN group was similar to that for salivary NO_3_^−^ concentrations with a significant difference between time points (*p* = 0.001). Compared with baseline, urinary NO_3_^−^ concentration increased significantly on the 1st and 3rd day (based on combined data obtained at week 4 and week 8) (Δ 3.9 mM/L, 95% CI: 1.5, 6.3 mM/L, *p* = 0.001) and (Δ 4.3 mM/L, 95% CI: 1.9, 5.9 mM/L, *p* < 0.001), respectively (Figure S5). There was a significant linear relationship between NO_3_^−^ dose and change from baseline in urinary NO_3_^−^ concentration (R^2^ = 0.46 *p* < 0.001, Figure 8).

#### 3.4.4. Salivary Nitrite Strips

**Figure 9:**
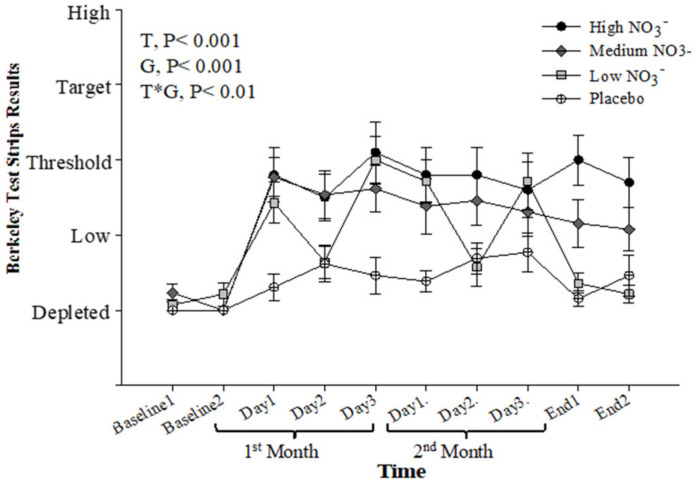
Salivary NO_2_^−^ strips readings (Berkeley). Mean salivary NO_2_^−^ strips readings (Berkeley) measured at baseline, 4 weeks, 8 weeks, and after 13 weeks. HN (High NO_3_^−^; two 70mL shots of BJ/day, morning and evening), MN (Medium NO_3_^−^; 70 mL of BJ/day), LN (Low NO_3_^−^; 70 mL of BJ every alternate days) and PL (placebo; 70 mL of NO_3_^−^ depleted BJ). Each shot of BJ contains 400 mg of NO_3_^−^. Data were analysed using a 2-factor repeated measures ANOVA (time*group of intervention). Data are expressed as mean ± SEM, (*n* = 50).

Baseline salivary NO_2_^−^ concentration, measured by the strips, was similar for all intervention groups (*p* > 0.05). Repeated-measure analysis showed a significant effect of time (*p* < 0.001), intervention (*p* < 0.001), and time*intervention interaction (*p* < 0.001). During the intervention, the HN and MN groups showed significant elevation in salivary NO_2_^−^ concentrations when compared with PL (*p* < 0.001 and *p* = 0.002, respectively). The LN group showed a more moderate increase in salivary NO_2_^−^ concentration (*p* = 0.05) with day-to-day variation mirroring the alternate day provision of the BJ supplement (Figure 9). There was a significant correlation (r = 0.41, *p* < 0.001) between salivary NO_2_^−^ concentration measured by the strips and that measured by ozone-based chemiluminescence (Figure S6).

### 3.5. The Relationship between the Duration of Postal Delivery Time and the Concentration of NO_2_^−^ and NO_3_^−^ Saliva and Urine

A sensitivity analysis was conducted to check whether the delay in receiving some samples affected the biomarker concentrations. Overall, there was no statistically significant difference in salivary NO_2_^−^ and NO_3_^−^ and urinary concentrations of NO_3_^−^ with time between posting by the participant and receipt of samples in the research centre (*p* > 0.05, Figure S7). Post hoc analysis showed a trend towards a lower salivary NO_2_^−^, but not salivary NO_3_^−,^ concentration, in samples received after 3 or more days compared with 2 or 1 days (*p* = 0.07 and *p* = 0.08, respectively).

### 3.6. Acceptability of Dietary Intervention and Study Protocol

Completed questionnaires were returned by 52 participants. The primary reasons for participating in the study were: (1) interest in nutritional research (*n* = 25) and (2) health benefits of beetroot (*n* = 18). Regarding their expectations from participating to the study, the majority of participants had no obvious expectations (*n* = 28) while some expected that the BJ could improve their health and wellbeing (*n* = 18). Interestingly, the majority of participants (*n* = 38) expressed an interest in being approached for a similar future study, whereas only 13 said that they would not participate in another similar study. The main reasons for not wishing to participate in a similar future study were (1) taste of BJ and (2) duration of the study. Other participants would not participate because of the side effects and/or the saliva collection procedure. One participant said: “We would definitely taste the product first!” In addition, almost half of the participants (n = 24) reported that they would not join a longer study (i.e., with a duration longer than three months).

Thirty-three participants reported that they did not eat or drink BJ regularly and the most frequent reasons were the limited availability and its unpleasant taste: “I don’t like beetroot at all”. “I like beetroot but it’s not always available and convenient to prepare”. When participants were asked whether or not they would recommend BJ, the overall responses (*n* = 34) were supportive and some of the key reasons are outlined below:

“I believe it provides benefits to blood circulation.”

“I feel my thoughts are clearer. My focus on things is better. I even took it to Amsterdam with me!”

“It depends on research results; I would not recommend it due to the taste.”

“I believe it’s good for health and it has a pleasant taste!”

“I felt my memory slightly improved and my blood pressure was down.”

“It’s a convenient way to take the beetroot, even though the taste is odd.”

“I don’t feel that many people would tolerate the taste without a proven benefit!”

“It has possibly improved my blood. Even the nurse was surprised from my blood results!”

The majority of participants reported no major concerns or preferences with the consumption of BJ while others found it difficult due primarily to taste:

“I hate the taste. The only way was to hold my nose whilst drinking.”

“Sometimes I had to hold my nose to gulp it down.”

“The smell, the syrupy texture, the Saltiness, all not good.”

“The taste made me gag.”

When participants were asked if they felt any difference as a result of the BJ consumption, several participants reported that they felt some beneficial effects after the intervention:

“Maybe an increase in my concentration levels.”

“My blood pressure reading has gone down, which is good.”

“Memory improvement.”

“Yes, I felt my cognition has improved, I am finding solutions to complicated situations!”

“I feel more alert.”

“Feel like I am thinking quicker.”

“I feel my memory improved.”

Overall, feedback about the measurements undertaken as part of the study protocol was positive and participants found the procedures acceptable. The only exception was for use of Intake24 to record dietary intake, where the feedback was mixed. Although 31 participants did not have any difficulty using Intake24, 19 found that Intake24 was difficult and complex to complete. The main problems were that it was time consuming and participants could not find all food items that they had eaten within the platform’s database.

## 4. Discussion

Over the past decade, there has been increased investigation of the beneficial effects of dietary NO_3_^−^ on health outcomes, including blood pressure, glucose control, insulin resistance, dyslipidemia, cognition, heart failure and peripheral arterial diseases [22,23,24,25,26]. However, most of these studies have been of short duration and there has been little investigation of the long-term effects of dietary NO_3_^−^ supplementation on physiological functions, especially in older people. To the best of our knowledge, this pilot RCT is one of the longest that examined the feasibility and acceptability of supplementation with different doses of NO_3_^−^-rich BJ in overweight and obese older adults.

### 4.1. Recruitment and Attrition

While the recruitment target was met, 19% of the participants did not complete the trial. Losses to follow-up are common in long-term dietary interventions and other studies have reported attrition rates of up to 21% in a 12-week intervention study [27]. The 3-month duration of our trial appeared to impact the attrition rate, as some participants found it difficult to maintain their adherence to the intervention for such a prolonged period. Specifically, some participants (*n* = 5) were able to consume BJ for up to, but not beyond, 6 weeks. Our study indicates that consumption of HN (2 BJ shots/day) may not be appropriate in long-term intervention (>13 weeks) as this group showed the highest attrition rate (38%), which suggests that this dose may not be acceptable for a significant proportion of older people.

Reasons reported by participants for dropping out of the study included having adverse effects, such as gastrointestinal (GI) symptoms (*n* = 3), or an unwillingness to ingest the BJ due to its taste or smell (*n* = 3). However, these adverse events were reported mainly from participants in the HN groups who consumed two BJ shots per day. Mild gastrointestinal symptoms have been reported within 2–2.5 h after ingestion of similar BJ doses (140 mL) [28,29]. This indicates that the consumption of concentrated BJ for prolonged periods may have adverse effects on gastrointestinal function in some individuals. In addition, the perceived unpleasant smell and taste of BJ is an important aspect of study design to consider in future investigations using a similar BJ product. One possible solution would be to introduce a taste session of the BJ product at the screening visit so that participants can make an informed decision on the acceptability of the product [30].

### 4.2. Compliance with Dietary Intervention

The challenge of ensuring adequate compliance in dietary intervention studies is well known, especially when such studies take place over longer periods and in free-living settings [10,31]. Although the present study was demanding, as reported by several participants, this study demonstrated a high degree of compliance with BJ consumption (i.e., >90%). This is an important indicator of the feasibility of this 13-week randomized trial. The incentive given at the end of the study, in conjunction with the support provided by the research team and the reminders sent during the trial, may have contributed to the high compliance. Only one participant had a low compliance; this participant stopped taking the BJ in the final two weeks due to GI problems.

### 4.3. Collection of Biological Samples at Home and Transfer to the Research Centre by Post

Measuring nutritional intake biomarkers can help in monitoring and confirming compliance with dietary interventions, and several studies have used concentrations of NO_3_^−^ and NO_2_^−^ in biofluids as a measure of NO_3_^−^ intake compliance in RCTs [13,32]. Thus, biological samples, including urine, saliva and salivary NO_2_^−^ strips, were collected at home by participants during the study. Previous studies showed that requiring participants to travel to the research centre frequently to provide biological samples reduced adherence to the study protocol [33]. The number of collected samples required can become unwieldy for participants, leading to loss of follow up. These barriers can be overcome by the use of at home collection biological sample protocols [34]. The present study showed that participants had an excellent adherence in collecting biological samples at home, which indicate the acceptability of this procedure.

The process of the transportation of the samples from participants’ homes to research centre may affect sample integrity in bioanalysis. The ideal approach would be to collect the samples from the participants and place them on dry ice on the day of collection, then store them within a few hours in −20 °C freezers. However, the delivery of the samples by mail was considered a more pragmatic and realistic approach to reduce the burden on researchers and participants. We predicted that most of the samples would be delivered to the research centre within two days, ensuring the stability of the samples. This feasibility study demonstrated that most samples were delivered within 2 days, with only a few cases taking more than 5 days. During this delay, samples were at room temperature, which could affect the concentrations of NO_3_^−^ and NO_2_^−^.

However, there was little evidence that these conditions affected NO_3_^−^ and NO_2_^−^ concentrations. Our analysis indicates that salivary NO_3_^−^ or NO_2_^−^ concentrations were stable for at least 48 h following their collection for samples maintained at room temperature, whereas urinary NO_3_^−^ concentrations were stable for longer periods (3 to 4 days) at room temperature. This suggests that both home collection of saliva and urine samples may be used, with appropriate protocols for collection and delivery times, as biomarkers of NO_3_^−^ intake.

### 4.4. Biomarkers of Nitrate Intake after Prolonged Beetroot Juice (BJ) Consumption

#### 4.4.1. Plasma Nitrate and Nitrite

There was a significant linear increase in plasma NO_3_^−^ concentration with increasing dose of BJ consumed. Since the time between the last BJ dose and the blood collection was ~12–18 h, this suggests that higher consumption of BJ for a long period leads to NO_3_^−^ accumulation in the blood due to its long half-life (5–8 h) [35]. In contrast, increasing doses of BJ did not result in a linear increase in plasma NO_2_; the change in plasma NO_2_^−^ was highest with MN dose with no additional changes with the HN dose. It might be expected that the HN dose would produce the highest levels of plasma NO_2_^−^, mirroring the finding with plasma NO_3_^−^. The explanation for these different findings between plasma NO_2_^−^ and NO_3_^−^ is not clear, but it is possible that the conversion efficiency decreased over a long period with the highest BJ dose and this should be investigated in future studies. In the current study, plasma NO_3_^−^ and NO_2_^−^ concentrations were elevated ~2- and ~1.3-fold, respectively in the low NO_3_^−^ dose group although blood collection was performed ~36 h following the last shot of BJ. This is in agreement with Bondonno et al. (2015) [36], who found that plasma NO_3_^−^ and NO_2_^−^ returned to baseline concentrations after 2 days following 7 days’ consumption of high NO_3_^−^ BJ.

#### 4.4.2. Salivary Nitrate and Nitrite

Around 25% of the blood NO_3_^−^ pool is actively taken up by the salivary glands and concentrated in saliva so that salivary NO_3_^−^ concentrations are 10–20 times higher than plasma NO_3_^−^ concentrations [37]. Although salivary NO_3_^−^ concentrations were elevated throughout the study in those receiving supplemental NO_3_^−^, a linear dose-dependent response was not apparent and salivary concentrations of NO_3_^−^ were similar for MN and HN groups. In further investigation of the data, we found that the relatively large increase in salivary NO_3_^−^ concentration observed in the MN group was driven by only one participant who had a very high change value in salivary NO_3_^−^ compared to the others. When data for that participant were removed, there was a clear linear dose-dependent response in salivary NO_3_^−^ (Figure S8).

A proportion (~5% of ingested NO_3_^−^) of salivary NO_3_^−^ is converted to NO_2_^−^ by the action of oral facultative microflora [38] so that the concentration of NO_2_^−^ in saliva is dependent on both the NO_3_^−^ in saliva and the NO_3_^−^ reductase activity of the oral microflora. Salivary NO_2_^−^ concentrations increased with time in those given the HN and MN doses, compared with the PL. However, we observed a marked decrease in salivary NO_2_^−^ concentrations in the HN group at 13 weeks. A similar pattern of reduction, but to a lesser extent, was observed with the MN dose. The reason for this decrease is unclear. It is unlikely that this reduction is related to the capacity of active NO_3_^−^ transport to the saliva, since salivary NO_3_^−^ concentration was increased at this time (13 weeks). In addition, it is unlikely that this finding is related to the time of sample collection, which was performed ~12–18 h following the last ingestion, or due to overnight fasting before the collection, as suggested by Blekkenhorst et al. (2018). In this study, participants were asked to follow similar instructions at home when they collected samples during the first and second months of the study, and there were marked increases in salivary NO_2_^−^ concentration observed at these times, especially with the HN group. Recent studies have shown that dietary NO_3_^−^ supplementation alters the oral microbiome [39,40] and increases the oral pH [40,41,42]. Oral NO_3_^−^ reductase activity is highest at a pH of ~7.0 to 8.0, and falls with increasing acidity [43]. Studies to date have been of short duration and whether long-term NO_3_^−^ supplementation has a different effect on the oral microbiota or on oral pH remains unknown.

In the LN group, there were very marked fluctuations in both salivary NO_3_^−^ and NO_2_^−^ concentrations when BJ was consumed on alternate days and this was even more apparent when data from week 4 and week 8 were combined. When the saliva samples were collected at ~36 h post BJ ingestion, the salivary NO_3_^−^ and NO_2_^−^ concentrations were much lower than those collected ~12–18 h following the last BJ ingestion. This finding indicates that, following increased NO_3_^−^ intake, salivary concentrations of NO_3_^−^ and NO_2_^−^ remain elevated for a relatively limited period of time (~up to 18 h), even with prolonged intake. Thus frequent (daily) consumption of NO_3_^−^ is required to maintain elevated concentrations of these biomarkers.

#### 4.4.3. Salivary Nitrite Strips

In a previous study, we found that salivary NO_2_^−^ strips are a reliable method to detect changes in salivary NO_2_^−^ over a 5-h period after a single shot of BJ [44]. The present study provided an opportunity to determine whether such strips can detect changes in salivary NO_2_^−^ over a long period of supplementation with different doses of NO_3_^−^. We observed a significant moderate correlation between NO_2_^−^ concentrations measured by the strips and those estimated by chemiluminescence for samples collected 12–18 h following BJ ingestion. Our observation of no increase in salivary NO_2_^−^ using the strips for samples taken 36 h after the last BJ ingestion is consistent with our observation of no significant increase in salivary NO_2_^−^ concentration in the same samples when assayed by chemiluminescence. In conclusion, this evidence confirms that salivary NO_2_^−^ strips are a useful tool for monitoring NO_3_^−^ intake in chronic NO_3_^−^ intervention studies.

#### 4.4.4. Urinary Nitrate

Approximately 70% of ingested NO_3_^−^ is excreted as such in the urine and it is not known how the remaining amount is excreted [45,46]. In the present study, there was a significant linear increase in urinary NO_3_^−^ concentration with increasing BJ consumption. In addition, over the 13 weeks of the study, urinary NO_3_^−^ concentration within each treatment group remained generally constant which suggests that there was no adaptation in urinary nitrate excretion following sustained exposure to higher intakes. This is consistent with results reported by Berends et al. (2019) and Jajja et al. (2015), who showed that urinary nitrate excretion remained at similar elevated levels during periods of the NO_3_^−^ supplementation.

The majority of ingested NO_3_^−^ is excreted in urine during the following 24 h [47]. Previous studies have shown that urinary NO_3_^−^ concentration returned to baseline 24 h after NO_3_^−^ ingestion [48,49] which is consistent with our observation that urinary NO_3_^−^ concentration decreased markedly when samples were collected 36 h after BJ ingestion in the low-dose NO_3_^−^ group.

## 5. Conclusions

In summary, this feasibility study provided valuable information on how to overcome the challenges in recruiting and retaining older overweight/obese adults in a relatively long-term BJ supplementation study. The appropriate use of intensives and phone or email reminders served to optimise participant retention, to maximize the collection of biological samples and to ensure overall compliance with the protocol of the study. In addition, this study demonstrated the feasibility of conducting a longer-term BJ supplementation intervention (lasting 13 weeks) among older participants, which, overall, was well tolerated with no serious adverse events. However, the study indicates that the HN dose may not be acceptable to a significant proportion of older people in long-term-intervention studies. Finally, the findings from this study will help inform the design of larger and longer studies investigating the effects of NO_3_^−^-rich BJ on health outcomes in older people.

## Figures and Tables

**Table 1:** Baseline characteristics of the study participants including use of medications.

	All	HN	MN	LN	PL	*p*-Value
Characteristics						
Number	62	16	17	14	15	-
Gender, M/F	24/38	10/6	12/5	4/10	5/10	0.16
Age (years)	66.3 ± 3.7	64.7 ± 3.5	66.7 ± 4.2	67.3 ± 2.7	65.7 ± 3.9	0.16
Education (years)	15.3 ± 3.0	16.0 ± 3	15.7 ± 2.6	15.0 ± 3.1	14.6 ± 3.1	0.73
Body weight (kg)	84.9 ± 12.6	90.9 ± 13.4	84.6 ± 10.5	80.1 ± 12.5	83.9 ± 12.6	0.15
BMI (kg/m^2^)	30.3 ± 3.7	30.5 ± 3.6	30.5 ± 3.2	29.9 ± 3.4	30.3 ± 4.8	0.99
WC (cm)	102.4 ± 9.2	104.5 ± 10.3	100.6 ± 9.6	102.1 ± 8.9	102.6 ± 8.2	0.59
FM (kg)	32.4 ± 8.7	32.1 ± 8.7	34.5 ± 9.0	31.3 ± 7.2	31.5 ± 10.0	0.90
FM (%)	37.8 ± 7.8	35.2 ± 8.2	39.2 ± 7.8	39.3 ± 6.6	37.1 ± 8.1	0.41
TBW (kg)	38.6 ± 6.3	41.3 ± 7.4	38.6 ± 4.5	35.6 ± 6.3	38.8 ± 5.9	0.18
SBP (mm Hg)	135.1 ± 14.7	130.8 ± 12.0	136.1 ± 10.4	139.5 ± 13.2	134.1 ± 12.9	0.46
DBP (mm Hg)	76.9 ± 9.4	75.8 ± 9.7	77.3 ± 9.1	77.8 ± 8.1	76.9 ± 11.2	0.96
PA (METs/week)	3667 ± 5604	2741 ± 1522	3257 ± 1845	2262 ± 1933	6280 ± 10512	0.20
Medication use						
Antihypertensive	6 (9.8%)	1 (6%)	1 (6%)	1 (7%)	3 (20%)	-
Hormonal therapy Thyroxin Testosterone	9 (14.5%) 1 (1.6%)	3 (19%) 1	1 (6%) 0	4 (29%) 0	1 (7%) 0	- -
Antihistamine	1 (1.6%)	0	0	1 (7%)	0	-
Lipid lowering agents	10 (16%)	5 (31%)	2 (12%)	1 (7%)	2 (13%)	-
Vitamin D	3 (3%)	1 (6%)	2 (12%)	0	0	-
Aspirin	1 (1.6%)	0	0	0	1 (7%)	-
Corticosteroid inhalers	2 (3%)	0	1 (6%)	0	1 (7%)	-
No therapy	35 (56%)	10 (63%)	9 (53%)	7 (50%)	9 (60%)	-

M/F, male/female; BMI, body mass index; WC, waist circumference; FM, fat mass; FFM, fat-free mass; SBP, systolic blood pressure; DBP, diastolic blood pressure; PA, physical activity; PA, physical activity; data are expressed as mean ± standard deviation (SD). Medications are presented as n (%). *p* values are based on one-way analysis of variance (ANOVA), except for gender which were based on Chi square test. HN; High nitrate, MN; Medium nitrate, LN; Low nitrate, PL; Placebo.

**Table 2:** Primary reasons for withdrawal from study.

Reasons	*N* (%)	Specific Reasons Provided by Participants
Uncomfortable bowel movement	3 (25)	“The number of times I spent going to the toilet increased, I go to the toilet up to eight times a day, it is obvious that something in the supplement has a certain effect on me” “I drank the beetroot juice (BJ), within 15 min I had to rush to the lavatory with diarrhoea”. “I need to give up, BJ seems to be giving me extremely loose bowels and I have had to stop taking it”
Taste and smell of BJ	3 (25)	“The smell, taste and texture are all extremely unpleasant, leaving me with a feeling of nausea for a long time after taking the BJ (Sometimes hours)”. “I am so sorry I have to pull out, because of the horrible taste of BJ, I was cheating: can’t drink all of the bottle, don’t want to spoil your study” “There is a fundamental problem in that I can’t tolerate the juice. I hate the juice; the consistency makes me feel sick and it is way too sweet. Much as I love beetroot, I cannot drink this”.
Moving out of the area	1 (8)	“I regret to say due to an enormous amount of travelling I have had to endure recently, I will have to drop out from your research scheme”
Complicated study	2 (16)	“Sorry, but I find it all a chore, with travelling in and out of town. Sorry I would like to exit from trial”
Teeth problems	1 (8)	“Unfortunately, I do think I will need to stop the study. too much sugar in the juices which are spoiling my teeth, I often have headaches after I drink them”
Bereavement family	1 (8)	-
Mouthwash use	1 (8)	-

## Data Availability

The data presented in this study are available on request from the corresponding author. The data are not publicly available due to privacy concerns.

## Supplementary Materials

The following are available online at https://www.mdpi.com/2072-6643/13/3/769/s1, Figure S1: Compliance with the intervention, Figure S2: Distribution of number of days between samples being posted by the participants and receipt by the researcher, Figure S3: Number of dietary intake records completed by participants. Participants used Intake24 software to record their dietary intakes every two weeks during the trial, Figure S4: Mean of salivary NO_3_^−^ (A) and NO_2_^−^ (B) concentrations in LN group (low NO_3_^−^ dose; 70 mL of BJ every alternate days), Figure S5: Mean of urinary NO_3_^−^ concentrations in the LN group (low NO_3_^−^ dose; 70 mL of BJ every alternate days), Figure S6: Scatterplot of Pearson correlation between Berkeley salivary NO_2_^−^ strips readings and salivary NO_2_^−^ concentrations. Figure S7: Salivary NO_2_^−^, NO_3_^−^ and urinary NO_3_^−^ (C) concentrations. Feedback questionnaire. Supplemental methods. Figure S8: Mean of salivary NO_3_^−^ concentrations after removing data from one participant.
