# Supplementary material for: Acceptability and Feasibility of a 13-Week Pilot Randomised Controlled Trial Testing the Effects of Incremental Doses of Beetroot Juice in Overweight and Obese Older Adults
Source: Nutrients. 2021 Feb 26;13(3):769. doi: 10.3390/nu13030769 (PMC7996834; doi:10.3390/nu13030769)
Supplement: Supplementary file 1 [file nutrients-13-00769-s001.pdf]

Online supplementary material

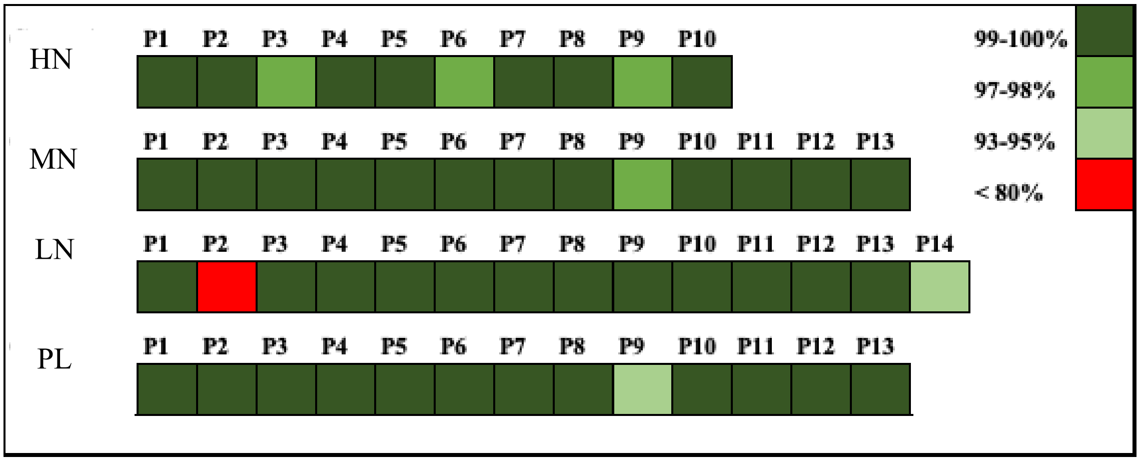

Figure S1: Compliance with the intervention.
Compliance with the intervention was assessed as the proportion (%) of BJ shots consumed by each participant in each intervention group. P; participant, HN; high NO_3^-, MN; Medium NO_3^-, LN; Low NO_3^-, PL; Placebo.

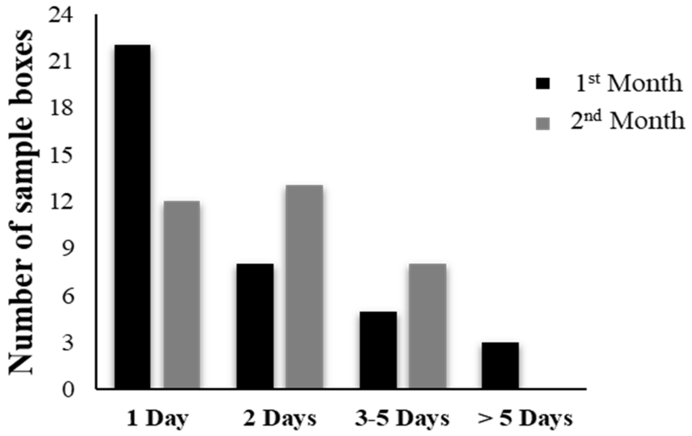

Figure S2: Distribution of number of days between samples being posted by the participants and receipt by the researcher.

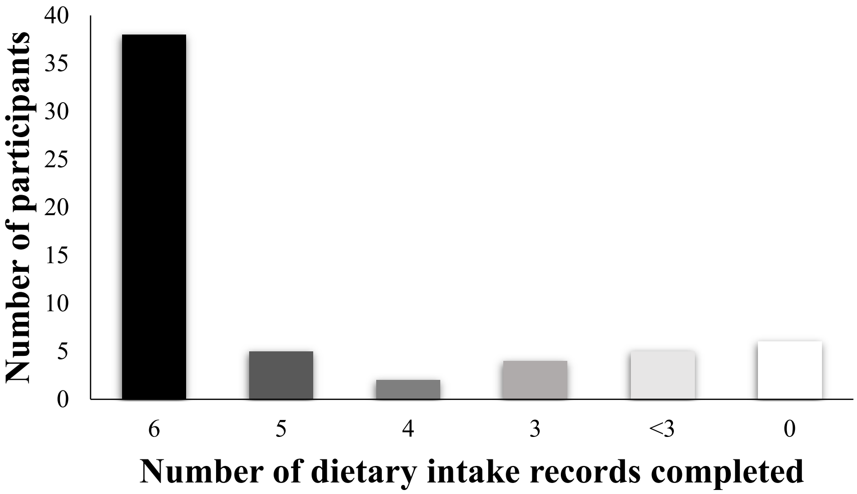

Figure S3: Number of dietary intake records completed by participants. Participants used Intake24 software to record their dietary intakes every two weeks during the trial.

A

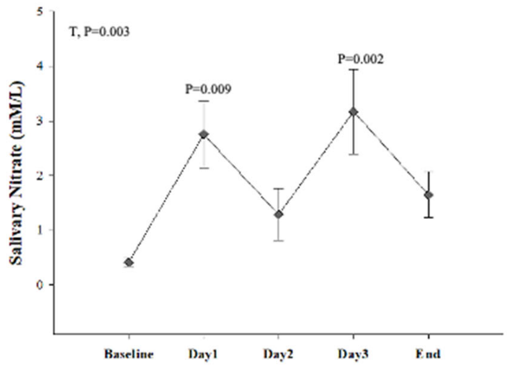

B

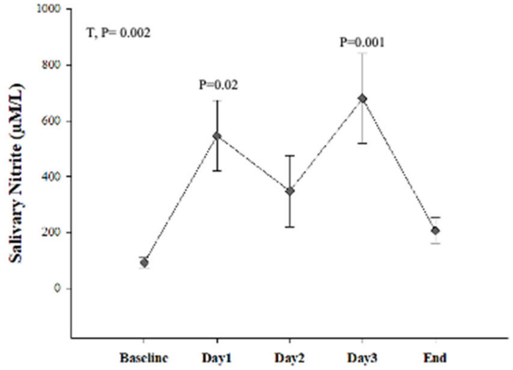

Figure S4: Mean of salivary NO_3^- (A) and NO_2^- (B) concentrations in LN group (low NO_3^- dose; 70 ml of BJ every alternate days).

Statistical analysis using one factor (time) repeated measure ANOVA. Saliva samples were collected on three consecutive days at week 4 and week 8. Data collected on the week4 and week 8 were combined. Data are expressed as mean ± SEM, (n = 50).

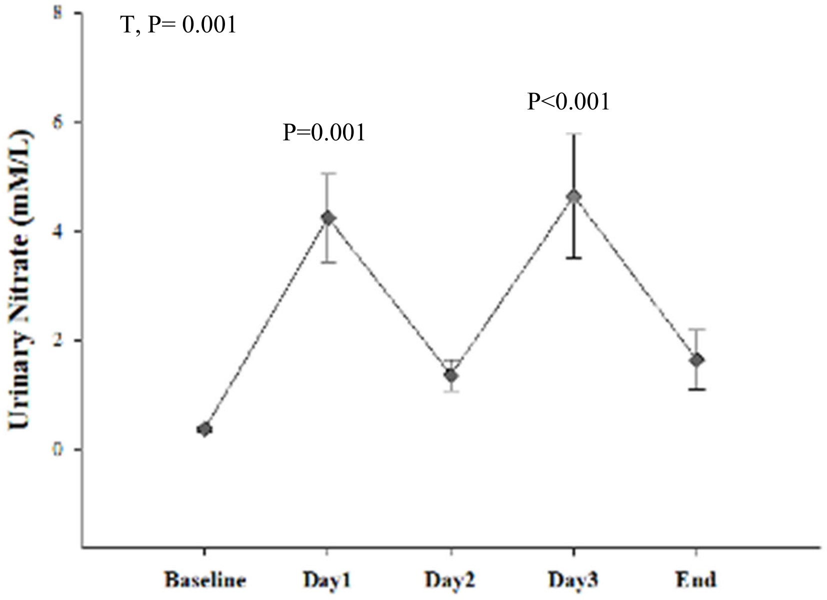

Figure S5: Mean of urinary NO_3^- concentrations in the LN group (low NO_3^- dose; 70 ml of BJ every alternate days).
Statistical analysis using one factor (time) repeated measure ANOVA. Urine samples were collected on three consecutive days at week 4 and week 8. Data collected on the week4 and week 8 were combined Data are expressed as mean ± SEM, (n = 50).

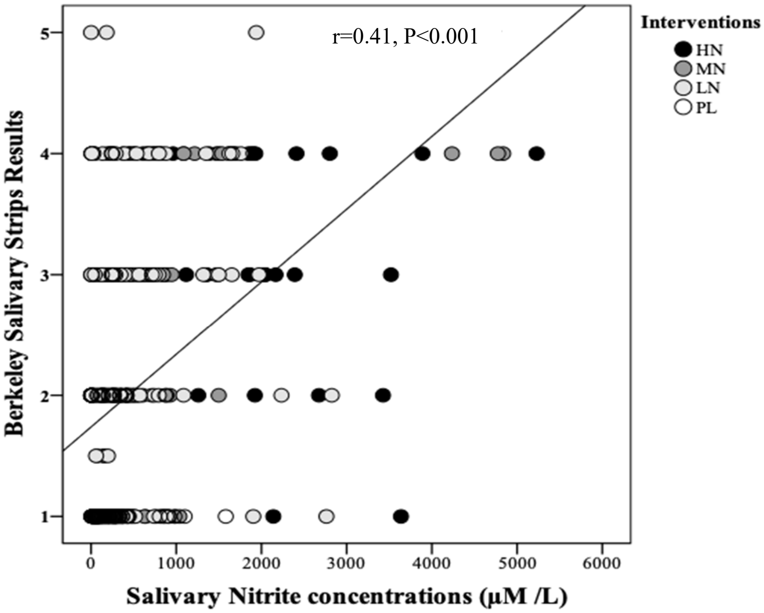

Figure S6: Scatterplot of Pearson correlation between Berkeley salivary NO_2^- strips readings and salivary NO_2^- concentrations.

Scatterplot showing Pearson correlation between Berkeley salivary NO_2^- strips readings and salivary NO_2^- concentrations measured by chemiluminescence; n=50. Berkeleysalivary strips results provided by the manufacturer (1=Depleted, 2=Low, 3=Threshold, 4=Target and 5=High).

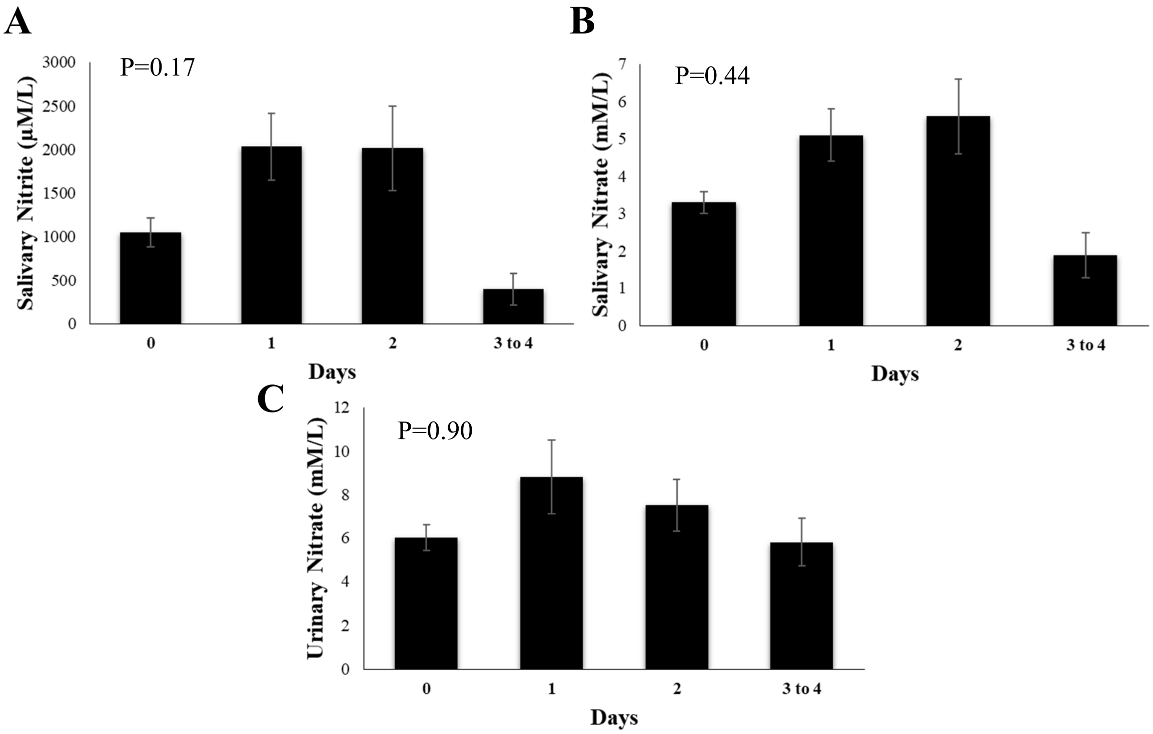

Figure S7: Salivary NO_2^- , NO_3^- and urinary NO3- (C) concentrations.

Mean salivary NO_2^- (A) and NO_3^- (B) and urinary NO_3^- (C) concentrations in samples received after a different number of days. Results presented in this analysis are based on combined data collected during the two interim monthly visits and across the 4 intervention groups. One-way ANOVA was used to test differences in biomarker concentrations between different days. Data are expressed as mean ± SEM.

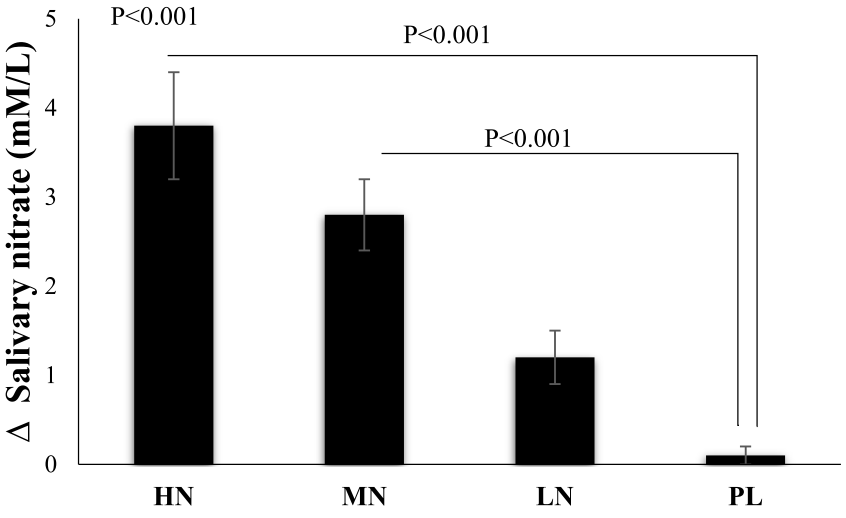

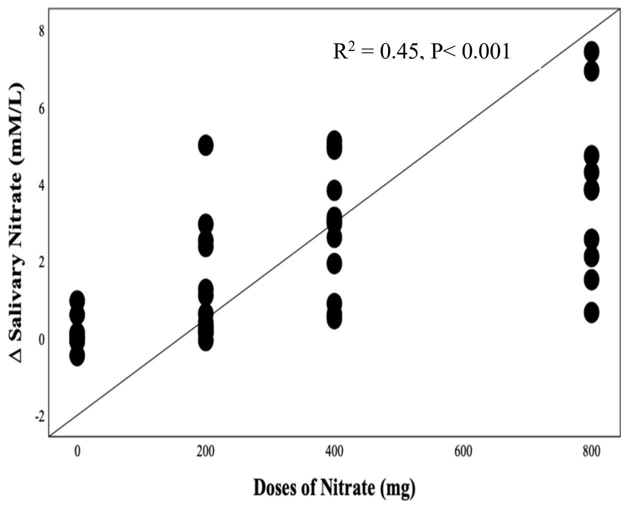

Figure S8: Mean of salivary NO_3^- concentrations after removing data from one participant.

Feedback Questionnaire

Nitrate, Cognition and Cerebral Blood Flow

Date: ________________ **ID:** __________________

1. **Why did you join this study?**

   ____

   ____

2. **What were your expectations from the study?** ______________________________

3. **Would you like to be approached for another study similar to this?**

   Yes ☐ No ☐

   If no, why?

   [1] Duration ☐

   [2] Beetroot juice taste ☐

   [3] Side Effects ☐

   [4] Other ____

   ____

4- **Would you join another study if it had a longer duration (>3months)?**

   Yes ☐ No ☐

   If yes, what is the longest duration of the study you could follow? _______months

Nutritional supplementation

1. **Do you eat or drink beetroot regularly?** Yes ☐ No ☐

   If yes, how many times a week? ____

____

If no, why? ____

____

2. **Would you recommend this product to a friend?** Yes ☐ No ☐
   If yes, why? ____

   ____

3. **Do you prefer having raw beetroot or still beetroot juice if you participating in another study?** Raw beetroot ☐ Beetroot juice ☐
   Why?____

   ____

4. **Which one of the following interventions did you follow?**
   [1] One bottle per day ☐
   [2] One bottle every 2 days ☐
   [3] 2bottles per day ☐

5. **Was it difficult to drink this dose of beetroot juice during the study?**
   Yes ☐ No ☐
   If Yes, please explain why? ____

   ____

6. **Have you felt any difference as a result of the beetroot juice intervention?**
   If so, please explain? ____

   ____

7. **If any, what was the most inconvenient aspect of taking the beetroot juice?**

   ____

   ____

8. **Would you drink beetroot juice regularly?** Yes ☐ No ☐
   If No, why?
   [1] Side effects ☐
   [2] Cost ☐

[3] Taste ☐

[4] Other ____________________

9. **Have you forgotten/missed any doses?** Yes ☐ No ☐

What was the reason? ____________________

____

Measurement protocols

1. **Was the ONT protocol (collection of saliva samples) easy to follow?**

Yes ☐ No ☐

If no, why?

[1] Too detailed ☐
[2] Too many samples required ☐
[3] Inconvenient ☐
[5] Fasting
[6] Other ____________________

____

2. **Did you have any difficulties with ONT collection?**

Yes ☐ No ☐

If yes, why?

[1] Chewing cotton balls ☐

[2] Collecting Saliva ☐

[3] Other ____________________

____

3. **Did you have any difficulties recording the time at which you took your beetroot juice every day?**

Yes ☐ No ☐

If yes, why? ____________________

____

4. **Did you have any difficulties recording your 24h recall diet and using the software (INTAKE 24) every two weeks?**

Yes ☐ No ☐

If yes, why? ____________________

____

5. **Was collecting saliva, urine and saliva strips every 4 weeks for 3 days acceptable for you?**

Yes ☐ No ☐

If No, why____________________

____

6. **Did you feel any difficulty to post them?**

Yes ☐ No ☐

If yes, explain why ____________________

____

7. **Did you find the general procedure of vascular assessment acceptable for you?**

Yes ☐ No ☐

If no, why? ____________________

____

8. **Did you find the general procedure of cerebral blood flow assessment acceptable for you?**

Yes ☐ No ☐

If no, why? ____________________

____

9. **Did you find the cognitive function assessment using COMPASS software acceptable for you?**

Yes ☐ No ☐

If no, why? ____________________

____

10. Would be acceptable for you if the tests to assess cognitive function last more than 30 minutes?

Yes ☐ No ☐

Study design

1. **Was the number of visits appropriate?**

   [1] Appropriate ☐ [2] Too many ☐

   If 2, please explain why: ____

____

2. What were the main factors which motivated you to participate and complete this study?

[1] Commitment to the study

[2] Health benefits of beetroot

[3] Challenges of the cognitive function tasks

4 Interested in nutritional research

[5] Other ____

____

Do you have any other comment?

____

____

____

Thank you for your time!

Supplemental methods

Ozone-based chemiluminescence

Before analysis, plasma samples were deproteinised using cold ethanol that was first chilled to 0 °C. 250 µl of each plasma sample was placed in a 1.5 ml microcentrifuge tube and 500 µl of cold ethanol was added and the tubes were then vortexed for 10 seconds. The tubes were then centrifuged at 14,000 RPM for 5 minutes. The supernatant was removed to a pre-labelled tube. Urine and saliva samples were diluted to 1:100 in deionized water prior to the analysis. Deproteinised plasma and diluted saliva samples were used for the determination of NO_3^- and NO_2^- , while diluted urine samples were used for the determination of NO_3^- by ozone based chemiluminescence (NOA 280i, Analytix, UK). The standard curve for both NO_3^- and NO_2^- was created to calculate the concentrations of NO_3^- and NO_2^-. The diluted samples were injected into the purge vessel with an injection volume ranging from 10 µl to 100 µl. The quality of the peaks was checked visually. Samples were re-diluted with a higher dilution factor (1:20) if no peaks were detected. The area under the curve of each peak was used to calculate the concentration of NO_3^- and NO_2^-. Samples were analysed in singlicate and analyses were repeated for samples with low quality peaks. The quality of peaks was checked visually based on height, fronting and tailing. The calculations were based on the AUCs calculated automatically for each peak by the software which minimise the risk of between-operator differences in the calculation of the results.

Salivary nitrite strips

Berkeley strips (Berkeley Test®, CA, USA) were used as per the manufacturer's guidelines. Specifically, participants were asked to place the test strip with the 'saliva here' side on the tongue and swab it over a 10 s period covering different areas including the dorsal surface of the tongue. The two ends of the strip were folded and pressed gently for 10 s. The colour of the NO test pad was then allowed to develop over a 45 s period. The intensity of the colour was compared with a colour chart included in the product package (Depleted, Low, Threshold, Target and High), with darker colours corresponding to higher NO_2^- concentrations. For the purpose of quantitative analysis, categorical colours were given a categorical number from 1 to 5, where 1 corresponding to "Depleted" and 5 corresponding to "High". Participants were asked to repeat a similar procedure at home as required by the study protocol.
